# Mistimed malaria parasites re‐synchronize with host feeding‐fasting rhythms by shortening the duration of intra‐erythrocytic development

**DOI:** 10.1111/pim.12898

**Published:** 2021-11-22

**Authors:** Aidan J. O’Donnell, Megan A. Greischar, Sarah E. Reece

**Affiliations:** ^1^ Institute of Evolutionary Biology, and Institute of Immunology and Infection Research School of Biological Sciences University of Edinburgh Edinburgh UK; ^2^ Department of Ecology and Evolutionary Biology Cornell University Ithaca New York USA

**Keywords:** asexual replication, biological rhythm, circadian, fitness, intra‐erythrocytic development cycle, periodicity, *Plasmodium*

## Abstract

**Aims:**

Malaria parasites exhibit daily rhythms in the intra‐erythrocytic development cycle (IDC) that underpins asexual replication in the blood. The IDC schedule is aligned with the timing of host feeding‐fasting rhythms. When the IDC schedule is perturbed to become mismatched to host rhythms, it readily reschedules but it is not known how.

**Methods:**

We intensively follow four groups of infections that have different temporal alignments between host rhythms and the IDC schedule for 10 days, before and after the peak in asexual densities. We compare how the duration, synchrony and timing of the IDC differs between parasites in control infections and those forced to reschedule by 12 hours and ask whether the density of parasites affects the rescheduling process.

**Results and conclusions:**

Our experiments reveal parasites shorten the IDC duration by 2–3 hours to become realigned to host feeding‐fasting rhythms with 5–6 days, in a density‐independent manner. Furthermore, parasites are able to reschedule without significant fitness costs for them or their hosts. Understanding the extent of, and limits on, plasticity in the IDC schedule may reveal targets for novel interventions, such as drugs to disrupt IDC regulation and preventing IDC dormancy conferring tolerance to existing drugs.

## 1 INTRODUCTION

Biological rhythms enable organisms to undertake activities at the time of day they are best undertaken. For example, cycles of activity and rest occur in relation to the day‐night cycles driven by the 24 hourly rotation of the earth, in the manners that minimize exposure to predators or harsh environmental conditions or maximize mating opportunities.^1^, ^2^, ^3^ Biological rhythms are also important in the context of infections.^4^, ^5^ Many aspects of host immunity oscillate with a daily rhythm^6^, ^7^ and diverse parasites align activities to daily rhythms in transmission opportunities,^8^ resource availability^9^ and host defences.^10^ For example, transmission stages of filarial nematodes, including *Wuchereria bancrofti*, migrate from host tissues to the peripheral capillaries in a periodic manner to coincide with daily rhythms in the biting activity of their mosquito vectors^8^ and *Schistosoma* spp. cercariae emerge from their intermediate snail host in the early morning or evening, depending on whether the next host in their lifecycle is nocturnal or diurnal.^11^, ^12^ Scheduling transmission activities extend beyond coordinating with vector and host rhythms; daily rhythms in the environment also impose opportunities and constraints on transmission. For example, the sporulation of oocysts produced by *Isospora spp*. is reduced by UV light exposure and so, oocysts are shed in the host's faeces in the afternoon and evening.^13^, ^14^ Once inside a host, parasites are subjected to the full gamut of the host's rhythms, including cellular and molecular processes, physiologies and metabolism and its behaviours. For example, host circadian clocks control cellular processes that influence the success of viral entry into cells and dissemination through tissues, for SARS‐CoV‐2,^15^ hepatitis B^16^ and influenza.^17^ Viruses do not appear to have rhythms in their own activities but instead may manipulate host rhythms to facilitate replication.^5^, ^17^ Daily rhythms in the feeding‐fasting cycles of hosts appear to drive periodicity in the gene expression patterns of *Trypanosoma brucei* ^9^ and *Schistosoma mansoni*,^18^ as well as setting the timing of blood stage replication by *Plasmodium* spp. (malaria parasites).^19^, ^20^, ^21^

Intuition suggests the diverse rhythms documented in parasites should enhance fitness via between‐host transmission and/or within‐host survival. Across all pathogenic organisms, rhythms in malaria parasites are currently the best understood; from evolutionary and ecological perspectives to their molecular underpinnings.^4^ Malaria parasites exhibit rhythms lasting a multiple of 24 hours in the intra‐erythrocytic development cycle (IDC) which underpins asexual replication in the vertebrate host's red blood cells.^22^, ^23^ Specifically, malaria parasites develop synchronously throughout the IDC and burst to release progeny at a particular time of day which generates fever with a 24, 48, or 72 hour periodicity that characterizes malaria infection by different *Plasmodium spp*. For *Plasmodium chabaudi*, over 57% of the transcriptome is rhythmic, the IDC exhibits 24h periodicity and culminates at the end of the hosts feeding period.^19^, ^20^, ^24^, ^25^ Whilst the timing of host feeding‐fasting and metabolic rhythms are ultimately determined by the host's clock, the host's canonical transcription‐translation feed‐back loop (TTFL) clock does not directly affect the IDC schedule.^19^ Instead, the timing of transitions between the developmental stages of the IDC directly follows feeding‐fasting rhythms, with rhythmicity in the amino acid isoleucine fulfilling the criteria to act as a time cue.^26^ Coordinating the IDC schedule with host rhythms is important for parasite fitness. When the timing of the IDC schedule is out of synchrony with the host, parasites suffer losses in the number of both asexually replicating stages and sexual transmission stages^27^, ^28^, ^29^ are more vulnerable to antimalarial drug treatment,^30^ and gene expression patterns underpinning key cellular processes are significantly altered.^25^ Thus, *P*. *chabaudi's* IDC schedule allows parasites to maximally exploit rhythmicity in the resources they require from the host's food.^20^ Conveniently, this schedule also ensures the maturation of sexual stages coincides with the time‐of‐day vectors forage for blood.^31^

How the IDC schedule is aligned with host rhythms is mysterious. Parasites may simply be intrinsically arrhythmic yet benefit from rhythms imposed upon them by the rhythms of hosts/vectors. For example, perhaps mistimed IDC stages starve and die because host rhythms create an environment in which only certain stages survive at certain times of day. Most evidence suggests that malaria parasites (at least in large part) control their timing.^4^, ^25^, ^32^, ^33^, ^34^ This includes observations that *P*. *falciparum* can undergo dormancy during the IDC to survive antimalarial drug treatment,^35^ *P*. *chabaudi* controls its IDC duration via the gene, Serpentine Receptor Ten^25^ and both *P*. *chabaudi* and *P*. *falciparum* use a cue with a daily rhythm (isoleucine) to break IDC dormancy.^34^, ^36^ A key step in differentiating between the relative contributions of traits encoded by the genes of hosts vs parasites is to search for time‐keeping mechanisms in parasites. The components of clocks driven by TTFLs have been identified in the fungal pathogen *Botrytis cinerea* and its clock is used to schedule the expression of virulence genes.^10^, ^37^ However, there is little homology in the genes underpinning canonical circadian oscillators across divergent taxa, complicating the search for ‘clock genes’ in novel organisms.^38^, ^39^, ^40^ Further, parasites may keep time with simpler ‘reactionary’ strategies rather than circadian clocks (which confer the additional abilities of temperature compensation and anticipation), or via oscillators that pre‐date the TTFL.^4^, ^41^, ^42^ Gene expression rhythms in trypanosomes and malaria parasites do fulfil some of the phenotypic criteria of endogenous TTFL‐driven oscillators.^9^, ^25^, ^29^, ^32^, ^33^, ^43^

Given the importance of timing the IDC schedule correctly coupled with parasites’ likely ability to keep time, it is not surprising that when the timing of the IDC schedule is perturbed, parasites readily reschedule. For example, *P*. *chabaudi* recovers from a 12‐hour mismatch to the host's feeding‐fasting rhythm within 5–7 IDCs.^19^, ^29^ During natural infections, parasites may benefit from a time‐keeping ability if egress from the liver to initiate blood stage replication occurs asynchronously or at a different time of day to optimal for IDC stages. Here, we ask how plasticity (flexibility) in *P*. *chabaudi's* IDC schedule allows malaria parasites coordinate with host rhythms. Following a 12‐hour mismatch to host rhythms, we test whether rescheduling of the IDC involves parasite development speeding up or slowing down, and we examine the consequences of rescheduling for synchrony, timing and replication dynamics. Determining how the IDC schedule responds to mismatch required tracking infections over at least 7 days with samples collected every few hours. However, after several days of intensive sampling regimes, host rhythms become perturbed which has knock‐on consequences for parasite rhythms.^44^ To overcome this issue, we set up multiple cohorts by infecting mice a day apart such that mice in each cohort were sampled simultaneously only over a 24–28 hour window, with each cohort contributing data for a different day post infection.

We made no *a priori* predictions for how the IDC should reschedule due to contradictory observations in the literature, including that (i) closely related species have shorter (and asynchronous) IDC durations (22–23 hours for *P*. *berghei* and 18h for *P*. *yoelii* ^45^, ^46^) suggesting faster IDCs are biologically possible; (ii) the avian malaria *P*. *cathemerium*, appears to extend or reduce its IDC duration in response to different perturbations of host rhythms^47^, ^48^; (iii) the IDC is arrested in response to the loss of a putative timing cue^26^ suggesting mismatched parasites only need a 12‐hour pause to get back on time; and (iv) a 12‐hour mismatch means that the same amount of time must be recovered by either speeding up or slowing down, so taking (ii) and (iii) together, different parasites within and between infections may adopt opposite strategies, as suggested for *P*. *brasilianum*.^43^ Changes in the duration of the IDC could affect overall asexual replication in a number of non‐mutually exclusive ways. Intuitively, a shorter IDC should lead to faster replication over the course of infection, but this depends on whether speeding up comes with a cost of fewer progeny per parasite (ie fewer merozoites per schizont), or if lower ‘quality’ progeny arise from a mismatch to nutritional resources or less time overall to garner resources. Understanding the extent of, and limits on, plasticity in the IDC schedule is important because asexual replication is responsible for the severe symptoms of malaria and fuels the production of sexual transmission stages and conferring tolerance to antimalarials.^4^, ^35^

## 2 MATERIALS AND METHODS

We carried out a large‐scale experiment to investigate how the IDC reschedules to regain synchrony following different kinds of perturbation to the host's feeding‐fasting rhythm (‘rescheduling’), and a smaller repeat study to test whether parasite density influences the rescheduling process (‘dose dependency’).

### 2.1 Hosts and parasites

Hosts were either wild type (WT) C57BL/6J strain or *Per1*/*2*‐null clock‐disrupted mice previously backcrossed onto a C57BL/6J background for over 10 generations. *Per1*/*2*‐null mice lack genes (Period1 and Period2) that are integral for a functional core (TTLF) clock and as a result, are behaviourally arrhythmic (including feeding‐fasting patterns) when housed in constant darkness.^19^, ^49^, ^50^ Mice were mixed sexes, 8–10 weeks old, housed at 21°C, and given a standard RM3 pelleted diet (801700, SDS, UK) with unrestricted access to drinking water supplemented with 0.05% para‐aminobenzoic acid.^51^ All mice were allowed 2 weeks to acclimatize (‘entrain’) to their respective feeding‐fasting/light‐dark rhythms before being infected. *P*. *chabaudi* (clone DK) parasites were injected intravenously at a dose of 1 × 10^6^ parasitized RBCs for the rescheduling experiment or at either 1 × 10^5^ (low dose) or 1 × 10^7^ (high dose) parasitized RBCs for the test of dose dependency. To reduce any potential donor effects, inoculum consisted of a pooled mix of three donor mice given to all treatment groups within each cohort. All procedures were carried out in accordance with the UK Home Office regulations (Animals Scientific Procedures Act 1986; SI 2012/3039) and approved by the ethical review panel at the University of Edinburgh.

### 2.2 Experimental designs

**FIGURE 1:**
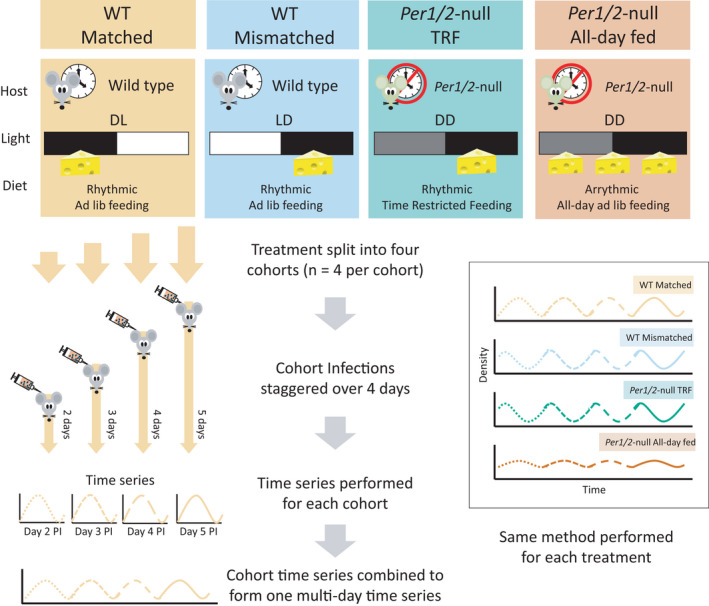
Experimental design. Four treatment groups were created from WT (C57BL/6J) or TTFL‐clock‐disrupted *Per1*/*2*‐null mice housed in a standard (LD) or reversed (DL) photoschedule, or constant darkness (DD), and given constant access to food (*ad lib* diet) or were fed with a time‐restricted diet (food available for only 12 hours per day; TRF). Mice from each of these groups were allocated to 4 cohorts (n = 4 each cohort per treatment). Cohorts within each treatment were infected over 4 subsequent days with ring stage parasites from WT donors entrained to a DL photoschedule. Thus, with respect to host feeding‐fasting rhythms, parasites in the matched treatment entered hosts in the same phase as their donor hosts (WT matched), parasites in the mismatched and TRF groups were ~12 hours out of phase to their hosts and must reschedule (WT mismatched & *Per1*/*2*‐null TRF), and parasites in the all‐day fed treatment entered arrhythmic hosts in which IDC rhythms become dampened (*Per1*/*2*‐null all‐day fed). All mice in all four cohorts were sampled on the same calendar day, every 4h for 28h, to cover the pre‐peak window of infections which spans days 2 to 6 post infection (PI), and cohorts 1–3 were sampled again (4h sampling for 24h) after a 3 day break to generate a post‐peak dataset covering days 7–10 PI. The time series for the cohorts with each treatment group were concatenated to generate pre‐peak and post‐peak time series for period estimates, whereas other rhythm parameters were estimated from individual infections

For the rescheduling experiment, wild type (WT) and *Per1*/*2*‐null mice were assigned to 4 treatment groups (n = 16 per group; Figure 1). The two WT treatments differed by their lighting regime (lights on 20:00–08:00 GMT (DL) and lights on 08:00–20:00 GMT (LD)) and were each provided with all‐day access to food (*ad libitum*). Mice in these groups followed their usual nocturnal feeding rhythms and fed primarily in their dark phases (08:00–20:00 GMT for the DL group and 20:00– 08:00 GMT for the LD group). The two groups of *Per1*/*2*‐null mice were housed in constant darkness (DD, with dim red LED) and provided with either a time‐restricted feeding diet (TRF) in which food was only available 21:00 to 09:00 GMT (analogous to the feeding window of the WT LD treatment) or was allowed all‐day access to food. Due to their arrhythmic behaviour, mice in the latter group feed continually throughout the 24h day.^19^ Note, TRF protocols differ from dietary restriction in that there are no weight loss implications of TRF (*Per1*/*2*‐null TRF mean ± SEM weight loss (g) for the 2 week entrainment period before infection = 0.1 ± 0.57).

Each of the four treatment groups were split into four cohorts (n = 4 mice per cohort per treatment) and infected with a synchronous population of ring stage parasites originating from donors housed in DL. This generated treatment groups in which parasites were matched to host feeding rhythms (WT matched); mismatched to host feeding rhythms and must reschedule by ~12 hours (WT mismatched and *Per1*/*2*‐null TRF); and infecting hosts without host feeding rhythms (*Per1*/*2*‐null all‐day fed). Cohorts were infected in a staggered design with the first cohort infected on day 5 followed by the other cohorts on days 4, 3 and 2. As a result, at any sampling time point, infections within each treatment group span 4 consecutive days post infection. Each cohort can therefore be concatenated to form a time series spanning multiple days.

To test whether the main experiment revealed general patterns for rescheduling or if the process depends on parasite density we compared how many IDC were required for parasites in WT mismatched infections initiated with two different doses (1 × 10^5^ and 1 × 10^7^ infected RBCs) to reschedule to the host's feeding‐fasting rhythm. Infections (n = 5 per cohort per dose) were initiated with the same staggered design for 4 cohorts as above and sampled every 4 hours for 32 hours from 08:00 GMT spanning day 2–6 PI.

### 2.3 Sampling and data collection

For the rescheduling experiment, mice were sampled at 4‐hourly intervals over two windows; for 28h to generate a pre‐peak window time series spanning days 2–6 PI, and for 24h to generate a post‐peak window time series spanning days 7–10 PI. The sampling regimes were set such that each cohort overlapped with the preceding/subsequent cohorts in terms of hours post infection (hpi). For the pre‐peak time series, each cohort overlapped by 2 sampling points, by a single sample overlap in the post‐peak time series, and by 3 sampling points for the dose experiment. These overlaps allowed us to determine if infections within each treatment were repeatable enough across cohorts to concatenate their data for some of the analyses. All mice contributed samples throughout the pre‐peak window for the main experiment (n = 4 per cohort) and the dose comparison (n = 5 per cohort). Five mice (2 from the *Per1*/*2*‐null TRF group and 3 from the *Per1*/*2*‐null all‐day fed group) were withdrawn from the experiment due to severe malaria symptoms following the pre‐peak window. This reduced the sample sizes in the *Per1*/*2*‐null TRF group to 3 for cohorts covering days 7–8 and 9–10 PI and in the *Per1*/*2*‐null all‐day fed group the cohorts covering days 8–9 and days 9–10 PI were reduced to 3 and 2 respectively. At each sampling point, a thin blood smear was taken to assess IDC stage distribution (from the proportion of parasites at each IDC stage in each smear) and RBC densities per ml were measured by flow cytometry (Z2 Coulter Counter, Beckman Coulter) immediately after sample collection. Blood smears were stained with 10% Giemsa for 12mins and IDC stages quantified based on parasite size, number of nuclei and the appearance of haemozoin, and summed to estimate total parasites.^19^, ^20^ Ring stage density per ml of blood was obtained from the product of the proportion of rings and RBC density.

### 2.4 Data analysis

Parasite densities at time‐points in which cohorts overlap were log‐transformed and compared between cohorts using either linear mixed‐effect models with mouse identity fitted as a random effect (pre‐peak and dose‐dependence datasets, due to multiple overlaps) or with GLMs (post‐peak dataset, due to one overlap). Parameters of rhythmicity (amplitude, phase, period) were determined using a maximum entropy spectral analysis (MESA).^52^ Infections for which MESA could successfully fit a rhythm between the period limits of 18–34 hours were classed as rhythmic. MESA was chosen because it is robust against baseline trends and large differences in amplitude across time that are characteristic of parasite density dynamics. Verification of MESA outputs was performed using Fast Fourier Transform Non‐linear Least Squares (FFT‐NLLS), Lomb‐Scargle and MetaCycle (Meta2d).^53^ Before rhythmicity analysis, ring densities were log‐transformed to reduce the exponential increase exhibited during infections. For the period analyses, additional baseline detrending via kernel smoothing was also performed (detrending was not necessary for amplitude and phase analyses). Rhythm amplitude and phase were determined from the time series data from each infection individually (time series length: pre‐peak window = 28 hour, post‐peak window = 24 hour). Amplitude is a unit‐less measure (denoted numerically between zero and one) representing the relative difference between maximum and minimum of an oscillation and was analysed with generalized linear models (GLMs). Phase represents peak timing of the oscillation (ie peak ring density) and was analysed with Bayesian circular GLMs. Period measures are best determined from longer time series with multiple cycles and therefore were calculated from datasets generated by averaging replicates within cohorts at each time point. Before period analysis, rhythmicity of the concatenated dataset was verified using the BD2 eJTK method. Parasite densities across hpi were compared between treatments in each infection window using linear mixed‐effect models with mouse identity nested within cohort as a random effect. RBC loss and weight loss were calculated for each cohort by subtracting the RBC/weight at the end of the time series from the beginning and were analysed using GLMs. For all models, to avoid overfitting due to small sample sizes ‘Akaike information criterion‐corrected’ (AICc) values were calculated, and a change in 2 AICc (ΔAICc  = 2) was chosen to select the most parsimonious model. Rhythmicity analysis, MESA, FFT‐NLLS and Lomb‐Scargle analyses were performed with Biodare2 (https://biodare2.ed.ac.uk/) ^54^ and all other analyses, including the Metacycle rhythmicity analysis, were performed with R v. 4.0.2 (R Foundation for Statistical Computing, Vienna, Austria).

## 3 RESULTS

### 3.1 Concatenating cohorts

**FIGURE 2:**
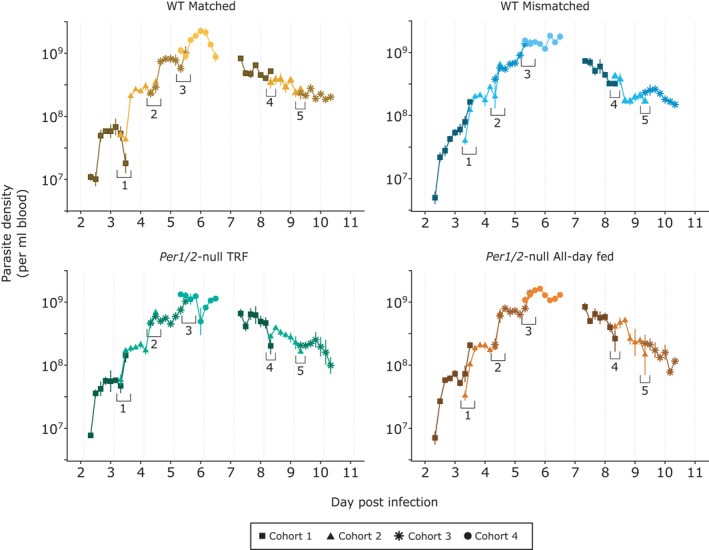
Mean ± SEM parasite density (per ml blood). Each cohort (represented by differing point shape and colour gradient) was comprised of replicate infections sampled over subsequent days post infection. Time points in which samples occurred at the same age of infection for each consecutive pair of cohorts are indicated by numbered brackets. Overlaps 1–3 occurred in the pre‐peak window and each consisted of 2 time points, whereas overlaps 4–5 each had single time point and occurred in the post‐peak window. Mice were either WT (C57BL/6J) or clock‐disrupted *Per1*/*2*‐null mice with parasites that were matched to the host's feeding‐fasting rhythm (WT matched), forced to reschedule to align with the host's feeding‐fasting rhythm (WT mismatched & *Per1*/*2*‐null TRF), or experienced arrhythmic hosts (*Per1*/*2*‐null all‐day fed). n = 4 infections per cohort for all groups in the pre‐peak window. For the post‐peak window, n = 4 for WT groups, n = 3–4 for *Per1*/*2*‐null TRF, and n = 2–4 for the *Per1*/*2*‐null all‐day fed group

To confirm that cohorts within each treatment are repeatable enough to represent longitudinally sampled infections, we compared parasite densities at the hours post infection (hpi) for which consecutive pairs of cohorts overlapped (Figure 2). For both the pre‐ and post‐peak window of the infections, incorporating cohort into the models did not improve model fits indicating that densities at these time points did not vary significantly between cohorts (Supplementary Information (SI) Table 1). Thus, we proceed with using data concatenated across cohorts for estimations of period and analyses of density dynamics. Whereas other characteristics of rhythms (amplitude, phase) can be calculated from the short time series for each individual infection.

**TABLE 1:** Rhythmicity analysis and measures of IDC period calculated from representative datasets (log^10^ ring stage density averaged across replicate infections contributing to each time point). For the rhythmicity analysis empirical‐JTK was performed and Benjamini Hochberg (BH) corrected *p* values are presented. For the period analysis, each dataset was analysed using Maximum Entropy Spectral Analysis (MESA, in bold) and results verified with Fast Fourier Transform Non‐linear Least Squares (FFT‐NLLS), Lomb‐Scargle and Metacycle (Meta2d). Each period estimates is accompanied by the model's goodness of fit (GoF; for which values close to zero indicate better fits), or for Meta2d, the BH corrected p value is appropriate. For the pre‐peak window, period was calculated using a 102h time series including four to five IDC cycles, and for the post‐peak window, period was calculated using a 72h dataset representing three IDC cycles. In both datasets mice were sampled every 4h

		IDC period (hours)							
		*MESA*		*FFT‐NLLS*		*Lomb‐Scargle*		*Meta2d*	
	Rhythmicity	Period	GoF	Period	GoF	Period	GoF	Period	*p*
*Pre‐peak window*									
WT matched	<0.0001	**23.4**	**0.29**	23.59 ± 0.63	0.32	23.58	0.28	23.84	<.0001
WT mismatched	<0.0001	**21.3**	**0.38**	21.38 ± 0.69	0.41	21.36	0.38	20.89	<.0001
*Per1*/*2*‐null TRF	<0.0001	**22.56**	**0.38**	22.45 ± 0.66	0.36	22.44	0.31	22.94	<.0001
*Per1*/*2*‐null all‐day fed	<0.0001	**22.5**	**0.26**	22.60 ± 0.59	0.29	22.6	0.25	23.03	<.0001
*Post‐peak window*									
WT matched	0.016	**23.34**	**0.67**	23.21 ± 2.41	0.58	23.32	0.54	23.77	<.0001
WT mismatched	0.001	**24.04**	**0.59**	24.00 ± 2.31	0.61	24.02	0.53	24.19	<.0001
*Per1*/*2*‐null TRF	0.003	**26.88**	**0.53**	27.30 ± 3.01	0.55	27.3	0.52	27.52	<.0001
*Per1*/*2*‐null all‐day fed	0.016	**27.9**	**0.55**	30.04 ± 4.91	0.65	29.98	0.61	30.7	<.0001

### 3.2 IDC rhythms during rescheduling

**FIGURE 3:**
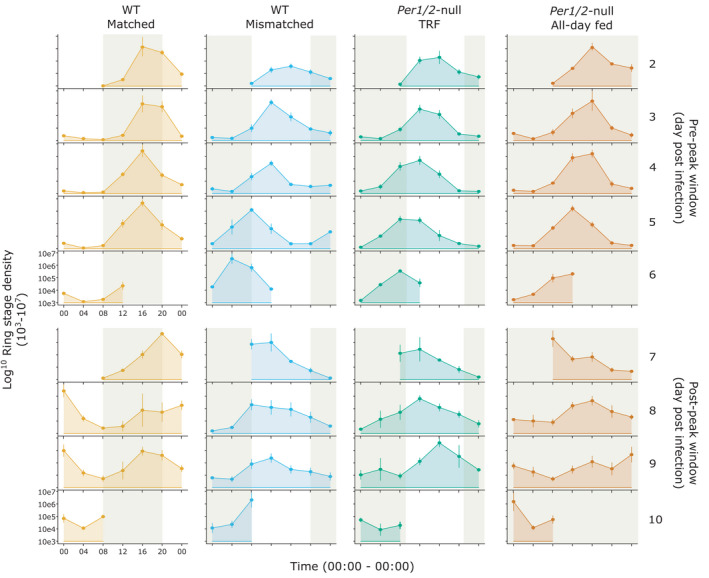
Mean ± SEM ring stage parasite density (per ml blood). Mice were either WT (C57BL/6J) or clock‐disrupted *Per1*/*2*‐null mice with parasites that were matched to the host's feeding‐fasting rhythm (WT matched), forced to reschedule to align with the host's feeding‐fasting rhythm (WT mismatched & *Per1*/*2*‐null TRF) or experienced arrhythmic hosts (*Per1*/*2*‐null all‐day fed). Shading represents the windows in which hosts fed and axes scales are identical across all plots. All infections including those without significant rhythms are included in the Mean ± SEM calculations: n = 4 infections per cohort for all groups in the pre‐peak window. For the post‐peak window, n = 4 for WT groups, n = 3–4 for *Per1*/*2*‐null TRF, and n = 2–4 for the *Per1*/*2*‐null all‐day fed group.

We focus on ring stages as a marker for the IDC schedule, as is usual for studies of *P*. *chabaudi* IDC rhythms^19^, ^20^, ^25^, ^28^, ^29^ (Figure 3; and Figure S1). In the pre‐peak window (days 2–6 PI) all infections exhibited rhythmicity in ring stage density except for a single infection in the WT matched treatment (from the days 2–3 cohort). In the post‐peak window (days 7–10 PI), 9/12 infections in both WT treatments were rhythmic, 8/10 infections were rhythmic in the *Per1*/*2*‐null TRF group and 6/9 infections were rhythmic in the *Per1*/*2*‐null all‐day fed group. All treatment groups in the concatenated time series were cyclic according to multiple approaches for assessing rhythmicity (Table 1). Because rhythmicity parameters can only be estimated for rhythmic infections, the non‐rhythmic infections were excluded for estimates of period, amplitude and phase.

#### 3.2.1 IDC duration (Period)

During the pre‐peak window, the concatenated time series reveal that periods were 1–2 hours shorter in the treatment groups with rescheduling parasites (WT mismatched = 21.30h, *Per1*/*2*‐null TRF = 22.56h) compared with infections matched to host feeding rhythms (WT matched = 23.40h; Table 1; Figure S2). Infections in hosts without feeding rhythms were also short (*Per1*/*2*‐null all‐day fed = 22.5h). The short period in rescheduling infections is evident by the five full peaks observed throughout the time series, whilst the WT matched infections had yet to reach the apex of peak five (Figure 3).

Post‐peak model fits were generally poorer compared with pre‐peak model fits but suggest that period remained close to 24 hours in WT matched infections (23.34h), lengthened by 3 hours to reach approximately 24 hours in the WT mismatched group (24.04h), and infections in *Per1*/*2*‐null hosts also extended by 3–5 hours to exceed 24 hours (*Per1*/*2*‐null TRF =26.88h, *Per1*/*2*‐null all‐day fed =27.90h; Table 1; Figure S3).

#### 3.2.2 IDC synchrony (amplitude)

**FIGURE 4:**
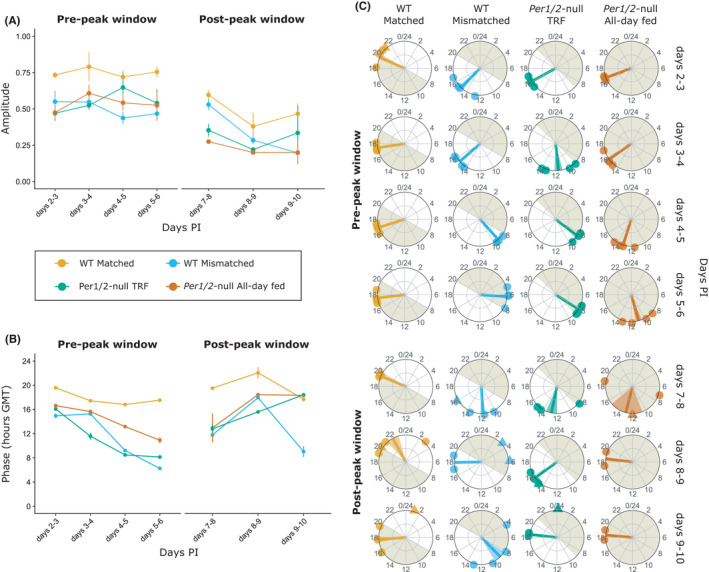
Mean ± SEM (A) ring stage amplitude and (B & C) peak ring stage phase calculated from ring stage density data for all rhythmic infections using a Maximum Entropy Spectral Analysis (MESA). In (C), mean peak ring stage phase is represented by a line with circular SD in shading. Circles and triangles represent phase estimates from infections classed as rhythmic and non‐rhythmic, respectively (the latter are omitted from (B) and do not influence mean ± SEM/SD but are included for completeness). Mice were either WT (C57BL/6J) or clock‐disrupted *Per1*/*2*‐null mice with parasites that were matched to the host's feeding‐fasting rhythm (WT Matched), forced to reschedule to align with the host's feeding‐fasting rhythm (WT Mismatched & *Per1*/*2*‐null TRF) or experienced arrhythmic hosts (*Per1*/*2*‐null all‐day fed). Shading represents the windows in which hosts fed. N = 4 infections per cohort for all groups in the pre‐peak window apart from WT matched (n = 3–4/cohort). For the post‐peak window, n = 2–4 for WT groups, n = 2–3 for *Per1*/*2*‐null TRF, and n = 2 for the *Per1*/*2*‐null all‐day fed group

We estimated rhythm amplitude (change between peak and trough for ring density) of each individual infection from its 28/24 hour time series (pre‐peak/post‐peak windows). During the pre‐peak window of infections (days 2–6 PI), amplitude is best described by the model containing only treatment as a main effect (ΔAICc  = 0, AICc weight = 0.94; Table S2). Specifically, parasites already coordinated with host feeding‐fasting rhythms (WT matched) had rhythm amplitudes ~50% higher than parasites in treatments causing rescheduling (WT mismatched and *Per1*/*2*‐null TRF) and in arrhythmic hosts (Figure 4a; Figure S4a; amplitude mean ± SEM: WT matched = 0.75 ± 0.03, WT mismatched = 0.50 ± 0.03, *Per1*/*2*‐null TRF = 0.55 ± 0.04, *Per1*/*2*‐null all‐day fed = 0.54 ± 0.03). Incorporating day PI reduced model fits (ΔAICc  = 5.49, weight = 0.06; Table S2) indicating that rhythm amplitude did not change significantly during the pre‐peak window.

Amplitude also varied during the post‐peak window of infection (days 7–10 PI) in a manner best explained by additive effects of day PI and treatment (ΔAICc  = 0, weight = 0.99; Table S2). Specifically, WT matched infections had the highest amplitude, 55% higher than rescheduling infections (WT mismatched and *Per1*/*2*‐null TRF) and ~200% higher than parasites in arrhythmic hosts (Figure 4a; amplitude mean ± SEM: matched = 0.48 ± 0.05, WT mismatched = 0.33 ± 0.06, *Per1*/*2*‐null TRF = 0.30 ± 0.04, *Per1*/*2*‐null all‐day fed = 0.23 ± 0.03). During the post‐peak window, amplitudes decreased from an average of 0.45 ± 0.04 on day 7 PI to 0.30 ± 0.05 on day 10 PI (Figure 4a; Figure S4a).

#### 3.2.3 IDC timing (phase)

We estimated the peak timing of ring density (‘phase marker’) for each individual infection from their 28/24 hour time series (pre‐peak/post‐peak windows). During the pre‐peak window of infections, phase is best explained by the interaction between treatment and day PI (Table S3). Around day 2 PI, the mean phases (hour GMT ± SD) are 19.58 ± 0.28 for WT matched infections, 14.95 ± 0.38 for WT mismatched, 16.07 ± 0.15 for *Per1*/*2*‐null TRF and 16.63 ± 0.07 for *Per1*/*2*‐null all‐day fed (Figure 4b,c; Figure 4b). For the WT matched infections, this timing aligns with the end of the host's feeding period (dark period) and donor infections, illustrating that these parasites have maintained their IDC rhythm. Peak phase in the rescheduling (WT mismatched and *Per1*/*2*‐null TRF groups) and *Per1*/*2*‐null all‐day fed groups had deviated by 3–5 hours, suggesting that whilst they were still mismatched to their new host's feeding‐fasting rhythm, rescheduling was underway.

As infections progressed, the phase of WT matched infections varied little, but the phase of rescheduling parasites (WT mismatched and *Per1*/*2*‐null TRF) diverged by approximately 10 hours to become aligned to host feeding‐fasting rhythms by day 6 PI (mean phase hour GMT ± SD: WT matched = 17.54 ± 0.18, WT mismatched = 6.26 ± 0.28, *Per1*/*2*‐null TRF = 8.14 ± 0.15). Infections in the *Per1*/*2*‐null all‐day fed group also deviated from the phase of the WT matched group with peak ring density at 10.92 h ± 0.46 GMT, five hours earlier than WT matched groups and 3–5 hours later than rescheduling groups. Overall, during the pre‐peak window, the mean rate of phase change for rescheduling infections was −2.77 ± 0.90 hours per day and a slower mean rate of phase change for the *Per1*/*2*‐null all‐day fed group of −1.90 ± 0.38 hours per day.

During the post‐peak window, the phase of peak ring density is also best explained by the interaction between treatment and days PI (Table S3). However, unlike in the pre‐peak window, phase change is not directional for all groups throughout the post‐peak window. Specifically, on day 7 PI the rescheduling infections and *Per1*/*2*‐null all‐day fed infections peak at a similar time, 7–8 hours earlier than WT Matched infections (Figure 4b,c; Figure S4b; mean phase hour GMT ± SD: WT matched = 19.52 ± 0.04, WT mismatched = 11.79 ± 0.36, *Per1*/*2*‐null TRF = 12.80 ± 0.78, *Per1*/*2*‐null all‐day fed = 12.97 ± 2.37). Phase became later in all groups by days 8–9 but patterns diverged by day 10 PI. Across days 7–10 PI, *Per1*/*2*‐null TRF and *Per1*/*2*‐null all‐day fed infections peaked at a similar time to WT matched infections (mean phase hour GMT ± SD: WT matched = 17.71 ± 0.40, *Per1*/*2*‐null TRF = 18.43 ± 0.05, *Per1*/*2*‐null all‐day fed = 18.34 ± 0.18), but the peak of WT mismatched infections became 9 hours earlier (9.04h ± 0.89 GMT) between days 8 and 10 PI.

### 3.3 Infective dose and rescheduling

**FIGURE 5:**
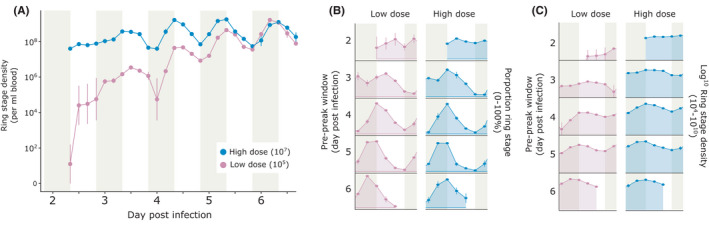
Mean ± SEM ring stage parasite density (per ml blood) for two parasite doses presented as (A) single time series and (B) proportion ring stage parasites presented as an actogram to correct for density differences and visualize change over sequential IDCs and (C) ring stage parasite density presented as an actogram. WT (C57BL/6J) mice in 4 cohorts and housed in LD received parasites from donors housed in DL at a low (1 × 10^5^ parasitized RBCs) or high dose (1 × 10^7^). Shading represents time at which hosts fed (night). Mice (n = 5 infections per cohort for each dose) were sampled every 4 hours for 32h starting at 08:00 GMT

In our second experiment, we compared the rates of IDC rescheduling by parasites in WT mismatched infections initiated with doses two orders of magnitude apart (10× higher and lower than the main rescheduling experiment). Asexual densities reflect the different infective doses, with low dose infections achieving a lower cumulative density than high dose infections (mean total cumulative parasite density ± SEM (×10^10^): low = 6.19 ± 0.14, high = 22.80 ± 0.08), and daily cumulative densities are best explained by the model with an interaction of days PI and dose (ΔAICc = 0, weight = 1; Figure 5a). Comparison of the overlaps revealed only minor (~3%) cohort differences between some infections in overlap 1 (Table S4), thus in concordance with the main rescheduling experiment, we concatenate cohorts to generate a single time series for each dose.

Resolution on the IDC schedule is low at the start of infections for the low dose because the fewer parasites that are used to initiate infections, the fewer that are observed for staging. Despite noisy date between days 2 and 3 PI in the low dose group, all infections exhibited very similar IDC rhythms during rescheduling (Figure 5). Once rescheduled to align with host feeding‐fasting rhythms, ring densities peak at the end of the feeding window (ie the right‐hand side of the shaded regions in Figure 5a,b) and both dose groups achieved this timing between days 5 and 6 PI. Specifically, mean phase hours (GMT) ± SD on day 6 PI were 6.36 ± 0.5 and 6.65 ± 0.21 for the low and high dose groups. The concatenated time series reveal that both low and high dose infections have periods of less than 24 hours (low dose = 21.52, high dose = 22.64). Further, both phase and amplitude do not differ (from day 3PI) between doses (amplitude is best explained by the null model (ΔAICc = 0, weight = 0.58; Table S5), and phase is best explained by the model containing days PI (Table S6).

### 3.4 Consequences of rescheduling for parasites and hosts

The design of the main rescheduling experiment enables us to examine whether different phase relationships between parasite and host rhythms influence the densities of asexual stages achieved over infections and the severity of symptoms experienced by hosts.

#### 3.4.1 Parasite performance

**FIGURE 6:**
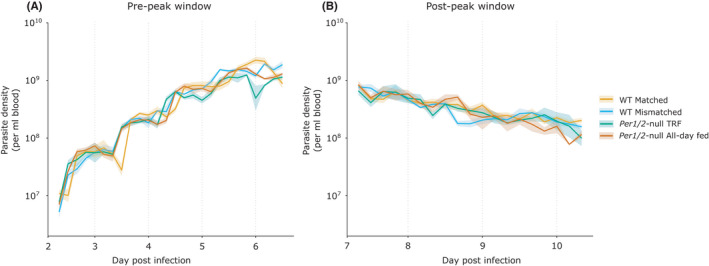
Mean ± SEM parasite densities (per ml blood) from concatenating cohorts. Mice were either WT (C57BL/6J) or clock‐disrupted *Per1*/*2*‐null mice with parasites that were matched to the host's feeding‐fasting rhythm (WT matched), forced to reschedule to align with the host's feeding‐fasting rhythm (WT mismatched & *Per1*/*2*‐null TRF) or experienced arrhythmic hosts (*Per1*/*2*‐null all‐day fed). (a) n = 4 infections per cohort for all groups in the pre‐peak window. (b) For the post‐peak window, n = 4 for WT groups, n = 3–4 for *Per1*/*2*‐null TRF, and n = 2–4 for the *Per1*/*2*‐null all‐day fed group. Sampling occurred every 4 hours starting at 08:00 GMT during the pre‐peak time series (days 2–6 PI) and also for the post‐peak time series (days 7–10 PI)

Parasite densities during both the pre‐ and post‐peak windows are best explained by day PI only (pre: ΔAICc = 0, weight = 0.997; post: ΔAICc = 0, weight = 0.997; Table S7) only. Including treatment reduced model fits (pre: ΔAICc = 11.70, weight = 0.003; post: ΔAICc = 11.53, weight = 0.003; Table S7), indicating that parasite densities during infections do not differ between treatments (Figure 6a,b).

#### 3.4.2 Disease severity

RBC loss during in the pre‐peak window of the infections was best explained by the additive effects of day PI and treatment (ΔAICc = 0, weight =0.96; Table S8; Figure S5a). *Per1*/*2*‐null TRF hosts experienced the greatest RBC loss (mean RBC loss ± SEM × 10^9^: 3.29 ± 0.41) followed by WT matched (2.66 ± 0.46) and *Per1*/*2*‐null all‐day fed (2.32 ± 0.39) hosts, whilst WT mismatched hosts experienced the least RBC loss (1.48 ± 0.32). Overall, hosts in all treatments lost ~2.0 ± 0.3 (×10^9^) RBCs daily across days 2–5 PI with greater loss occurring between days 5–6 PI (mean RBC loss ±SEM × 10^9^ = 3.83 ± 0.42). During the post‐peak window of the infections RBC loss was best explained by day PI alone (ΔAICc  = 0, weight = 0.95; Table S8; Figure S5b) as incorporating treatment reduced model fits (ΔAICc = 5.84, weight = 0.05). Because hosts recovered from anaemia during the post‐peak window, RBC switched from a loss of 0.89 ± 0.16 (mean ± SEM × 10^9^) on days 7–8 PI to a gain by days 9–10 (−0.56 ± 0.11).

Weight loss during both the pre‐ and post‐peak windows are best explained by day PI alone (pre: ΔAICc  = 0, weight = 0.46; post: ΔAICc  = 0, weight = 0.78; Table S8; Figure S5c,d). However, AICc model weights in these analyses are low (<50%) indicating high model selection uncertainty. Hosts experienced an average daily weight loss of 0.7 ± 0.08g during the pre‐peak window and loss was maintained during the post‐peak window (weight loss mean ± SEM (g): days 7–8 PI = 1.05 ± 0.31, days 8–9 PI = 0.61 ± 0.0.17) until days 9–10 PI when weight was gained (−0.45 ± 0.16).

## 4 DISCUSSION

Here, by analysing ~1200 samples, we demonstrate that phenotypic plasticity in the IDC duration allows *P*. *chabaudi* to recover from a ~12‐hour mismatch to host feeding‐fasting rhythms within approximately 5–6 days (Figures 3 and 4), in a manner independent of parasite density (Figure 5). Specifically, by speeding up (‘phase advancing’) each IDC by 2–3 hours, the timing of peak ring stages shifts within 5–6 IDCs to synchronize with the host's feeding‐fasting rhythm (Figure 4b,c). During rescheduling (ie WT mismatched and *Per1*/*2*‐null TRF groups), parasites experience minor reductions in synchrony (Figure 4a) but do not incur costs in terms of the densities achieved during either the pre‐ or post‐peak window of infections (Figure 6), and infecting an arrhythmic host (ie *Per1*/*2*‐null all‐day fed) does not impact parasite density (Figure 6).

Whilst our aim was to investigate the ecology surrounding rescheduling of the IDC, we also tested whether this process has longer‐term consequences throughout infections. We find that post peak, synchrony degrades in all groups (Figure 4a), the timing of peak ring density shifts or becomes more variable within groups (Figure 4b,c), and IDCs 3–4 became 3–4 hours longer in infections that had to reschedule (ie WT mismatched and *Per1*/*2*‐null TRF groups) and 6 hours longer in *Per1*/*2*‐null all‐day fed hosts (Table 1). Whilst the increased variability and dampening of rhythms in the post‐peak phase reduces confidence in the precision of period estimates, multiple approaches suggest that infections matched to host rhythms from the outset (WT matched) experienced the least disruption to period, synchrony and timing in the post‐peak window. During rescheduling, parasites exhibit altered transcriptional patterns associated with many important processes.^25^ Thus, stress experienced during rescheduling may have long‐term effects that render parasites more vulnerable to IDC disruption from the stress of host sickness. For hosts, their phase relationship with the parasite's IDC has a minor impact on virulence (Figure S5). Specifically, *Per1*/*2*‐null TRF hosts experience the most severe anaemia, losing approximately twice as many RBC as WT mismatched hosts, with *Per1*/*2*‐null all‐day fed and WT matched hosts experiencing an intermediate loss. However, these differences do not extend into the post‐peak phase and are not reflected in variation in weight loss, our other virulence measure. This suggests that hosts maintained similar relative levels of food intake across treatments and so, food levels and the impacts of sickness on host rhythms are not the sole drivers of the IDC schedule.

Intuition suggests there are several strategies that parasites could use to reschedule to a new host rhythm, including i) pausing IDC progression for ~12 hours; ii) undertaking an initial large phase shift within the first IDC, followed by fine‐tuning the schedule in subsequent IDC; iii) individual parasites within an infection employing different strategies, with some parasites speeding‐up and others slowing the IDC; or iv) changing the IDC duration by a fixed amount each cycle (faster or slower) and making linear progress to the correct alignment with host rhythms. Observing a single large shift in IDC timing (as predicted by option (i) or (ii)) could also be due to host rhythms imposing the IDC schedule by severe negative selection of mistimed IDC stages at a certain time of day. In contrast, exposure to a danger at a set time of day could not masquerade as options (iii) or (iv). That we do not observe a severe reduction in densities over a single IDC in WT mismatched and *Per1*/*2*‐null TRF infections, coupled with revealing malaria parasites adopt option (iv) demonstrates that parasites exert more control over their IDC schedule than negative selection by host rhythms. Why would parasites reschedule by changing the IDC duration by a fixed amount each cycle, and why is the period shortened by only 2–3 hours? Extending the IDC by 2–3 hours would align its schedule to host rhythms at the same rate but slowing down development or reducing overall replication rate might render parasites vulnerable to immune killing and delays building a source population for transmission stage production. Similarly, simply waiting for ~12hrs would incur a delay to replication. That we observe a 2–3 hr change in IDC duration is consistent with the recent discovery that loss of serpentine receptor 10 (SR10) causes *P*. *chabaudi's* IDC to speed up by ~2 hours.^25^ Perhaps parasites only express SR10 when in synchrony with host rhythms as a mechanism to maintain this schedule alignment? The IDC changed analogously in both types of rescheduling infection (WT mismatched and *Per1*/*2*‐null TRF); period estimates were similar for both groups and the phase of ring stages shifted to peak at the end of the feeding window, although the phase for WT mismatched parasites was more similar to the WT matched controls (Figure 4c). This could be because parasites can use timing information from additional rhythms operating in WT hosts. Alternatively, it might be optimal for ring stages to peak at the end of the feeding period, and parasites in *Per1*/*2*‐null TRF hosts can achieve this because they are not subjected to the potentially conflicting impacts of other host rhythms present in WT hosts.

Mismatch between the IDC schedule and host rhythms has been reported to reduce asexual replication rate and gametocyte densities during the pre‐peak window and also disrupt the expression patterns for genes involved in important cellular processes.^25^, ^28^, ^29^, ^55^ Parasites are thought to align to host rhythms to exploit rhythmic resources required from the host's food and to ensure transmission stages mature at the time‐of‐day mosquitoes seek blood meals.^31^ Thus, we expected the costs imposed by resource limitation starving certain mistimed stages plus any role of parasite‐parasite communication in rescheduling, being exacerbated at high densities and so, leading to the high dose infections rescheduling sooner. However, across both experiments with infective doses spanning 3 orders of magnitude, all parasites rescheduled via a 2–3 hour reduction in IDC duration and reached the same phase within 5–6 days PI. This suggests that regardless of circumstances, parasites are constrained to reschedule via a set reduction in the IDC duration. Such a strategy could be deployed without parasites needing to communicate, but it remains possible that cell‐cell communication^56^ is involved and that signals relating to how much the IDC duration should alter become saturating even at low density. Nonetheless, observing the same reduction in IDC duration across doses suggests this is the minimum duration for the IDC.

In contrast to previous studies,^28^, ^29^ we did not detect a reduction in overall asexual densities in mismatched compared with WT matched infections. Thus, why should parasites reschedule if there no apparent costs of mismatch? Whilst experimental designs here—notably sampling regimes and parasite genotypes used—differ with previous studies there are several other non‐mutually exclusive explanations. First, altered gene expression pattern of mismatched parasites^25^ suggests there are fitness consequences of the IDC schedule. Perhaps by altering cellular processes, parasites can compensate for costs of mismatch in well‐fed naïve hosts (as we used in here) because they are able to support parasites at any IDC stage throughout the circadian cycle. This scenario suggests that by establishing the correct IDC schedule early in infection, parasites are anticipating the need to mitigate against future resource limitation that would occur if mismatched and at high density in an ill host. Second, rescheduling must have a (hidden) negative impact on parasites to explain why a faster IDC does not lead to higher overall replication than for the WT matched controls. For instance, if the IDC is reduced by 2 hours, rescheduling parasites complete six IDCs 12 hours ahead of the WT matched parasites, but do not reach higher densities for the same age of infection. Perhaps, parasites trade‐off a faster IDC for a reduction in the number of quality of merozoites to maintain the same overall replication dynamics as parasites in control infections. Third, the ultimate selective driver for *P*. *chabaudi's* IDC schedule might be to coordinate transmission stage maturation with vector biting rhythms, and host‐feeding rhythms are a useful proxy for vector rhythms. These ideas could be tested by comparing matched and mismatched infections in hosts with different levels of physiological condition. Fourth, the *P*. *chabaudi* clone used here (DK) is less virulent than those used in previous studies^57^ and so may experience less severe costs of any resource limitation due to misalignment of the IDC.

We examined IDC rhythms in *Per1*/*2*‐null all‐day fed infections to establish how the IDC schedule is affected when parasites are neither mismatched nor exposed to time‐of‐day information. As expected, the IDC rhythm became dampened and its duration reduced, which may suggest a short‐free running period (if the IDC schedule is driven by an endogenous oscillator^32^, ^33^). Based on previous studies, we expected synchrony in *Per1*/*2*‐null all‐day fed infections to be eroded faster that we observed.^19^ Previous experiments followed parasites in singly housed mice, so it is possible that group‐housing in the present experiment maintained residual rhythms established by masking during the rearing of mice.^58^ Alternatively, other TTFL‐independent oscillators may be present in *Per*1/2‐null hosts, for instance, food‐anticipatory behaviours or non‐transcriptional oscillators that influence the IDC schedule.^19^ In keeping with a lack of overall costs to rescheduling parasites, infecting an arrhythmic host does not impact on asexual replication. However, exploiting an arrhythmic host might be best achieved by parasites without an IDC rhythm. Future work could examine whether parasites benefit from matching their IDC rhythmicity to the degree of rhythmicity their host exhibits.

Reflecting the lack of overt costs of perturbing the alignment of host and parasite rhythms on asexual density dynamics, we only observe minor differences in virulence between the groups. *Per1*/*2*‐null hosts tend to experience greater anaemia than WT hosts because mice deficient in *Per2* exhibit high susceptibility to acute erythrocyte stressors.^59^ However, anaemia dynamics are not related to whether parasites are rescheduling or experiencing dampened rhythms, do not extend into the post‐peak window, and weight loss does not vary between treatment groups. Thus, relative to the impacts of infection *per se*, the alignment of host and parasite rhythms appears inconsequential. Hosts experienced more severe symptoms during the post‐peak window (eg RBC densities drop to 20% of pre‐infection levels) and this likely explains the substantial variation in IDC rhythms in the post‐peak window. During the post‐peak window, IDC rhythms in all groups experienced substantial reductions in synchrony, variable phase changes and lengthened periods, although the WT matched group was least affected. The impacts of illness on host feeding behaviour coupled with dampened locomotor and temperature rhythms and ~1–3 h advancement of peak timing for these host rhythms^60^ may make it difficult for parasites to maintain an IDC schedule.

In summary, our experiments reveal that plasticity in the IDC schedule allows malaria parasites to reschedule following mismatch to host rhythms by reducing the IDC duration by 2–3 hours. This reduction in IDC duration might represent the minimal amount of time required to complete the IDC. The lower and upper limits of IDC duration are unknown but might be revealed by examining parasites in hosts with shorter or longer feeding‐fasting cycles. Neither parasites nor hosts experience significant short or long‐term consequences of perturbing the alignment between rhythms. However, some costs or trade‐offs appear to be involved in rescheduling because a faster IDC does not enhance overall asexual replication dynamics relative to matched parasites. This suggests parasites are able to maintain asexual densities whilst rescheduling, perhaps by trading IDC duration off against merozoite production.^61^ Such an ability to compensate might be expected to evolve if parasites often experience circumstances that require rescheduling, such as if egress from the liver is arrhythmic or occurs at a time of day misaligned to feeding‐fasting rhythms and highlights the importance of alignment with host rhythms for blood‐stages. Furthermore, there may be costs of rescheduling for transmission stage production, although rescheduling parasites do not appear to invest less in transmission.^55^ Understanding the extent of, and limits on, plasticity in the IDC schedule may reveal targets novel interventions, such as drugs to disrupt IDC regulation and preventing tolerance to existing drugs by IDC dormancy.

## AUTHOR CONTRIBUTIONS

AO’D and SR conceived and designed the project. AO’D carried out the experiments. All authors interpreted the data, prepared the manuscript and approved the final manuscript.

### PEER REVIEW

The peer review history for this article is available at https://publons.com/publon/10.1111/pim.12898.

## Supporting information

Supplementary Material

## Data Availability

The datasets supporting the conclusions of this article are available in the Edinburgh DataShare repository: https://doi.org/10.7488/ds/3065.
