# Supplementary material for: Mistimed malaria parasites re‐synchronize with host feeding‐fasting rhythms by shortening the duration of intra‐erythrocytic development
Source: Parasite Immunol. 2021 Nov 22;44(3):e12898. doi: 10.1111/pim.12898 (PMC9285586; doi:10.1111/pim.12898)
Supplement: Supplementary file 1 — Supplementary Material [file PIM-44-0-s001.docx]

# Supplementary Information:


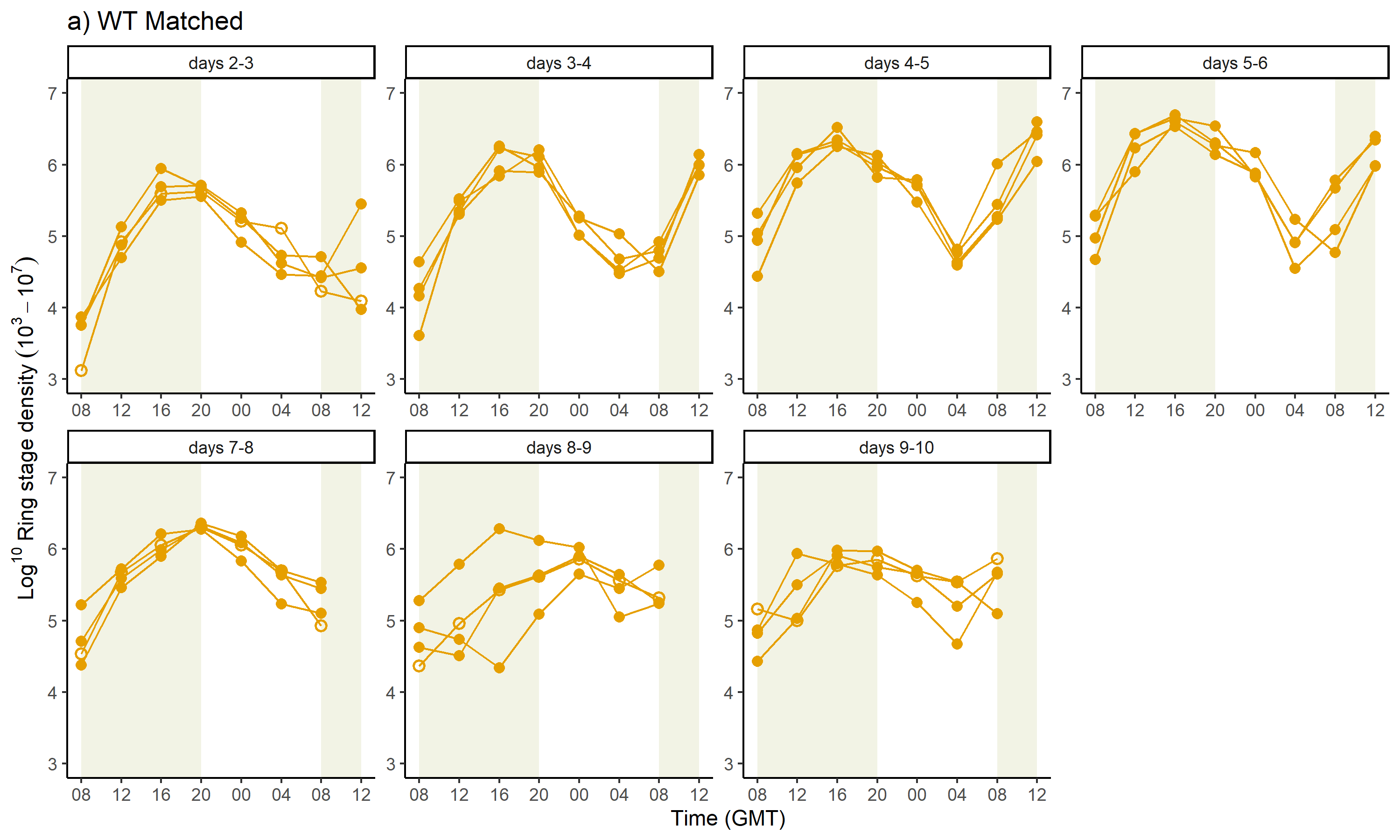


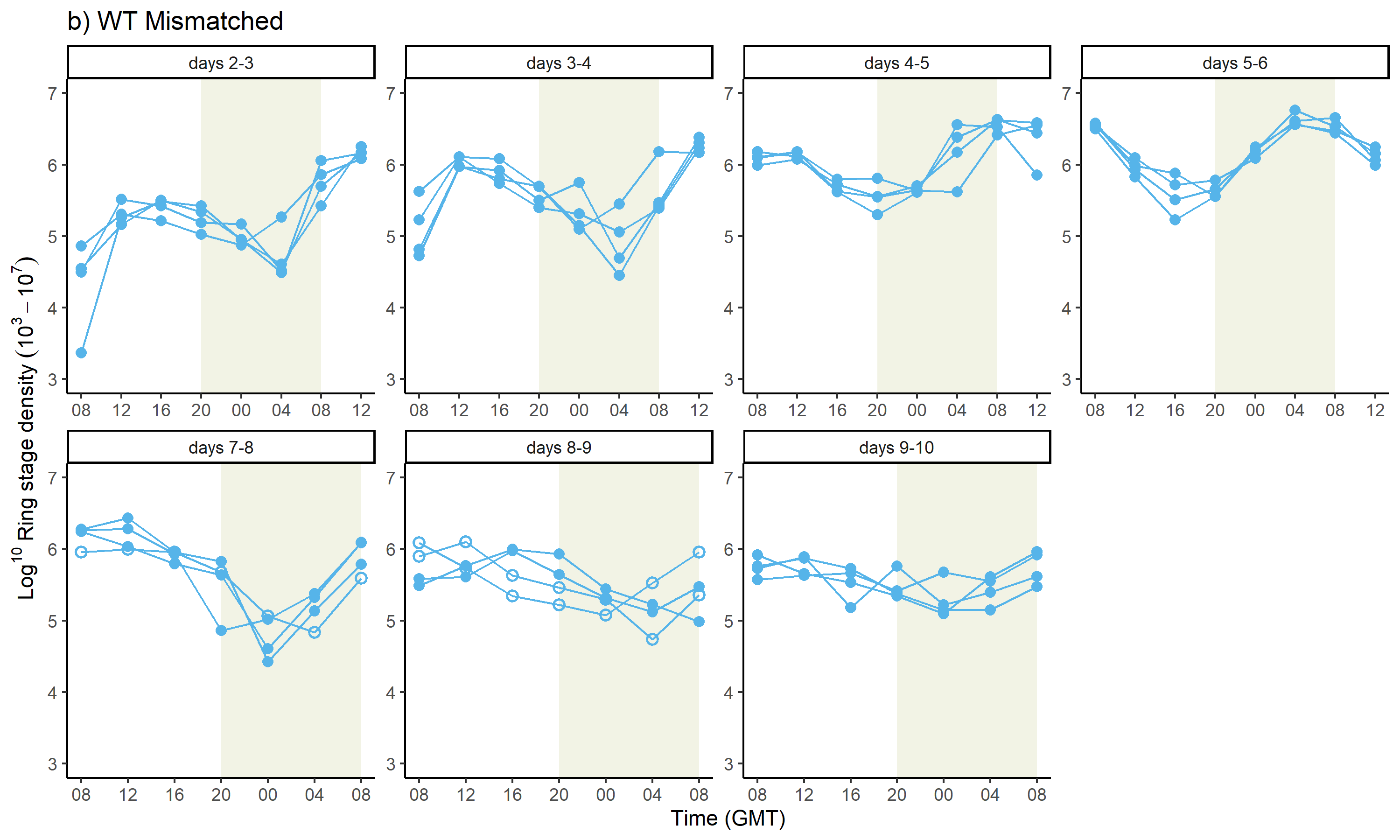


**
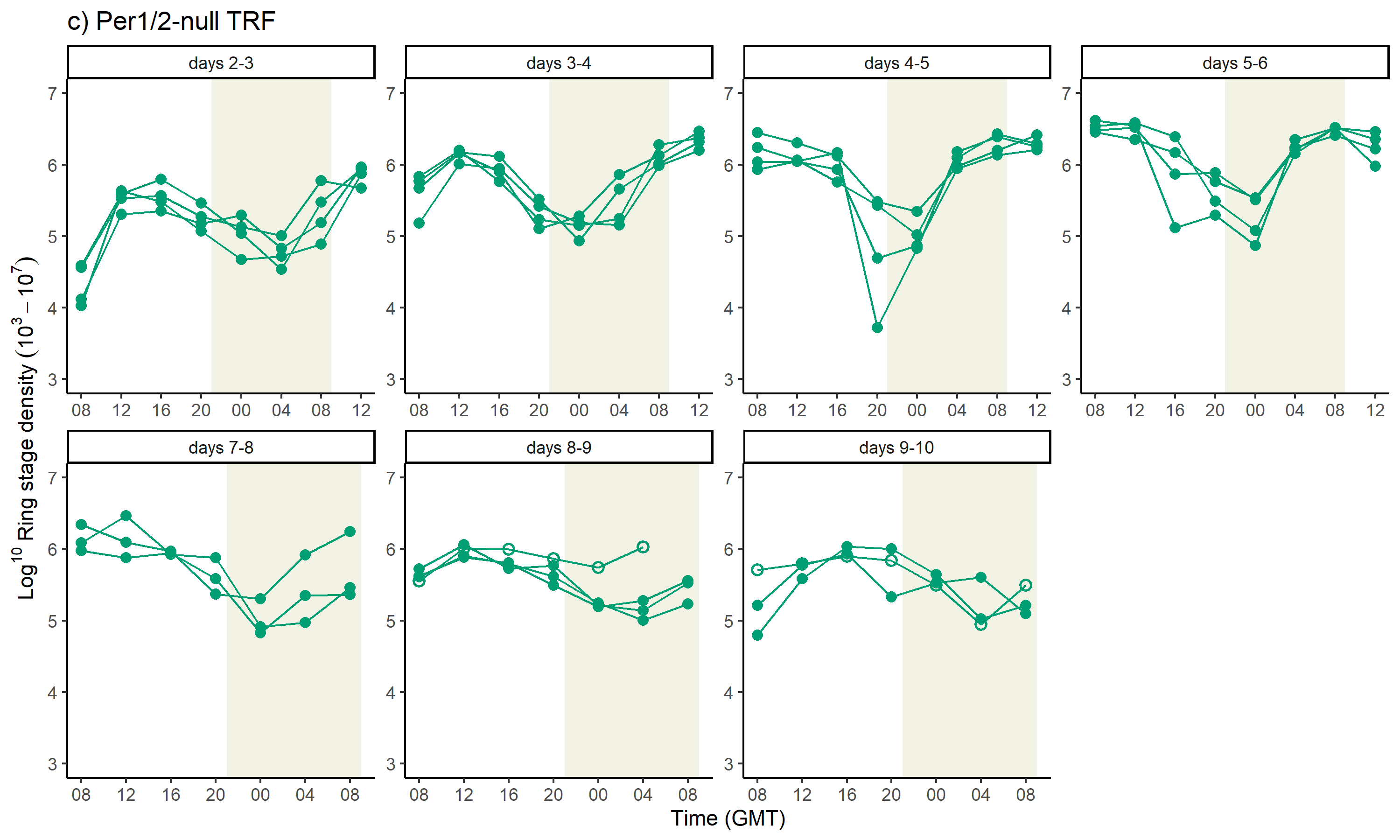
**

**
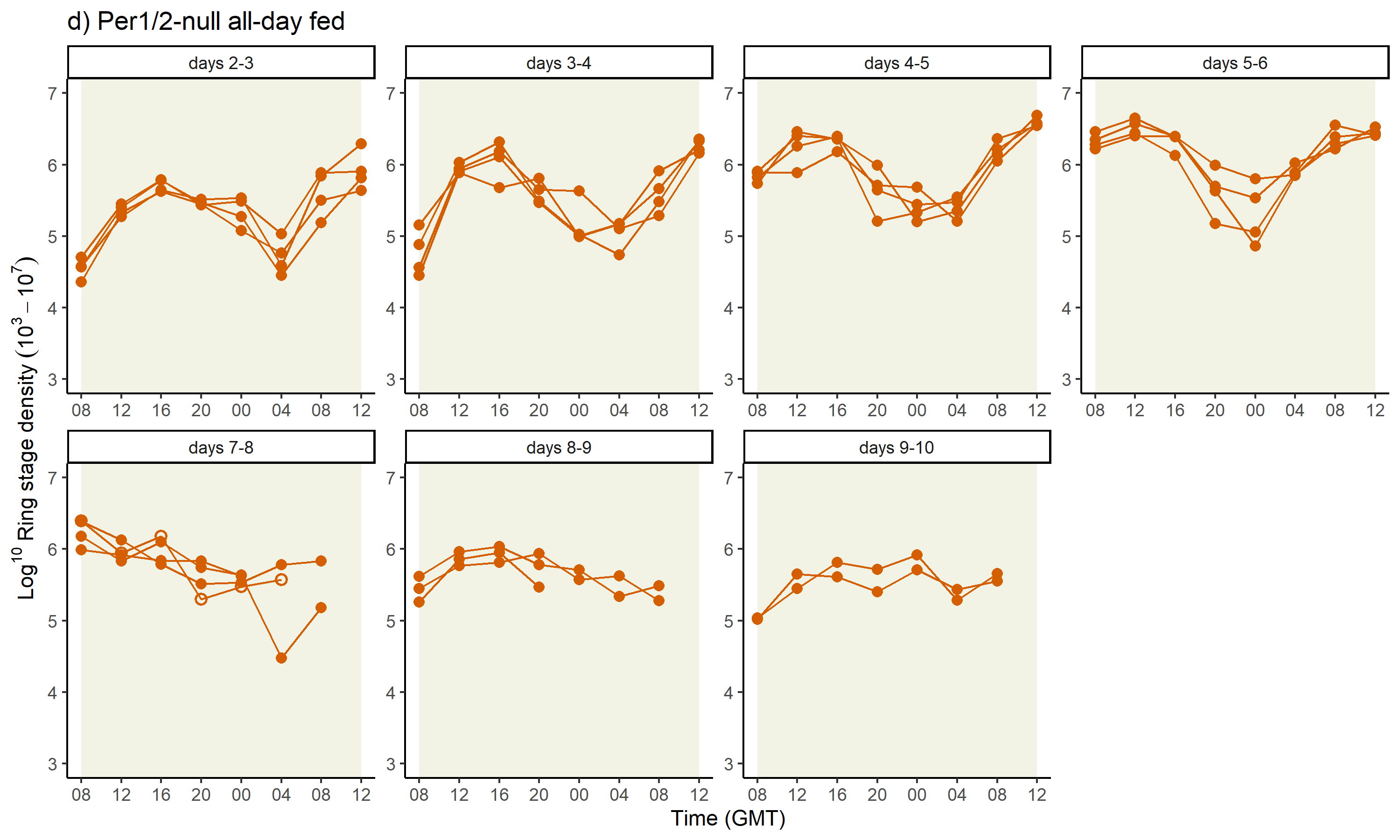
**

**SI Figure 1:** Ring stage parasite density (per ml blood) for each individual infection in the rescheduling experiment. Mice were either WT (C57BL/6J) or clock-disrupted *Per1/2*-null mice with parasites that: were matched to the host’s feeding-fasting rhythm (WT matched; panel a), forced to reschedule to align with the host’s feeding-fasting rhythm (WT mismatched & *Per1/2*-null TRF; panels b and c) or experienced arrhythmic hosts (*Per1/2*-null all-day fed; panel d). Open points represent infections that were not considered rhythmic and shading represents time at which hosts fed. Each plot represents a cohort. Days 2-6 are considered the pre-peak window of the infections, while days 7-10 are considered the post-peak window.


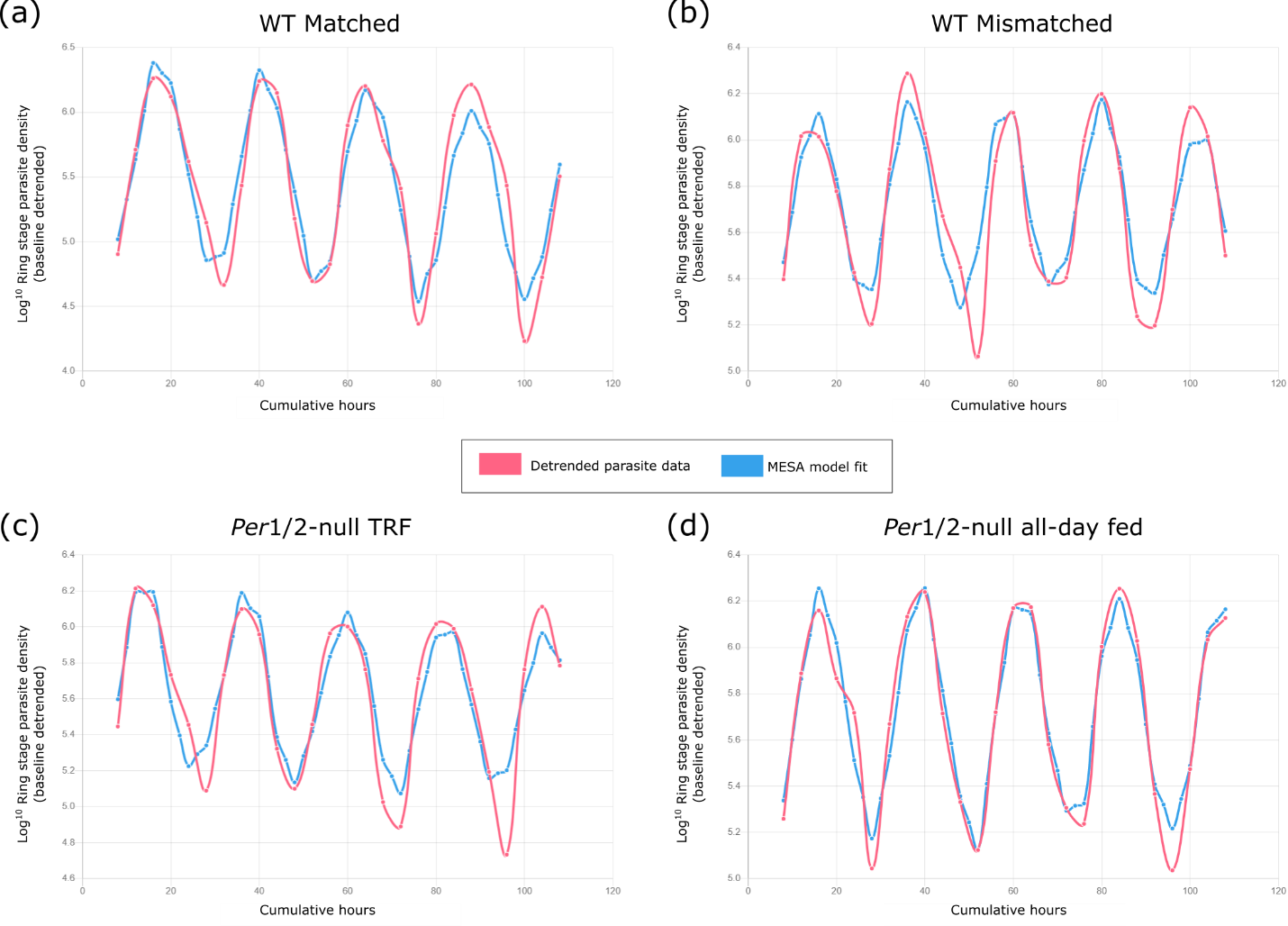


**SI Figure 2:** Maximum Entropy Spectral Analysis (MESA) model fits plotted against mean log_10_ ring stage parasite density (baseline detrended) from concatenated data during the Pre-peak infection window. Mice were either WT (C57BL/6J) or clock-disrupted *Per1/2*-null mice with parasites that: were matched to the host’s feeding-fasting rhythm (WT matched; panel a), forced to reschedule to align with the host’s feeding-fasting rhythm (WT mismatched & *Per1/2*-null TRF; panels b and c) or experienced arrhythmic hosts (*Per1/2*-null all-day fed; panel d).


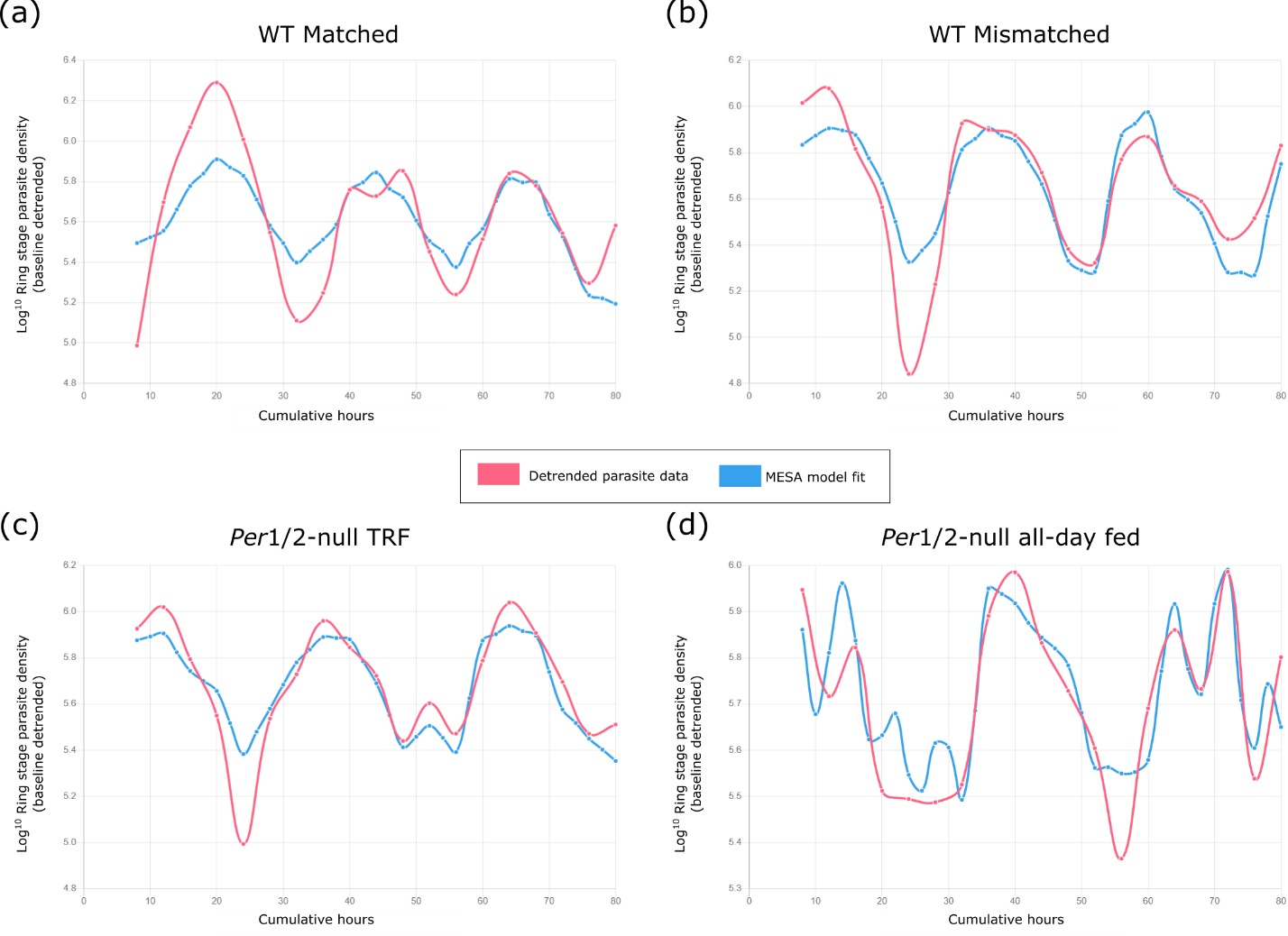


**SI Figure 3:** Maximum Entropy Spectral Analysis (MESA) model fits plotted against mean log^10^ ring stage parasite density (baseline detrended) from concatenated data during the Post-peak infection window. Mice were either WT (C57BL/6J) or clock-disrupted *Per1/2*-null mice with parasites that: were matched to the host’s feeding-fasting rhythm (WT matched; panel a), forced to reschedule to align with the host’s feeding-fasting rhythm (WT mismatched & *Per1/2*-null TRF; panels b and c) or experienced arrhythmic hosts (*Per1/2*-null all-day fed; panel d).


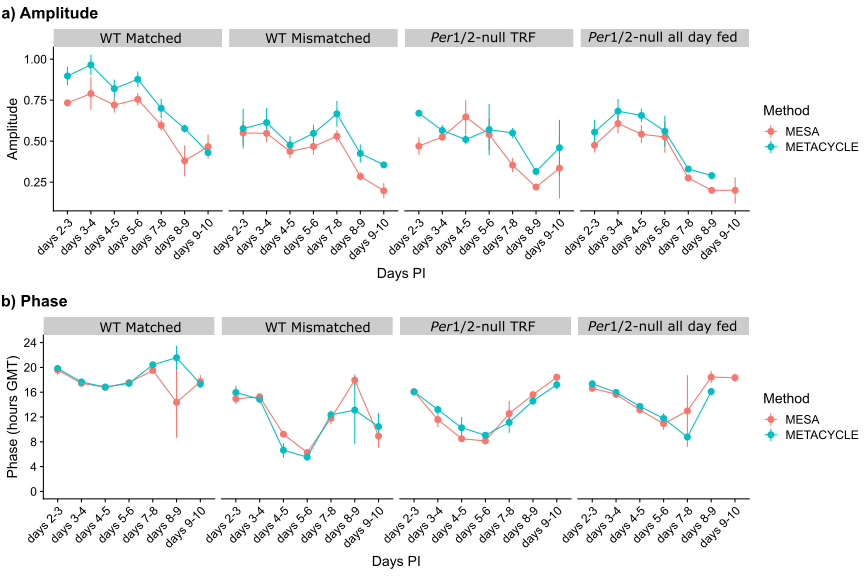


**SI Figure 4:** Mean ± SEM (a) ring stage amplitude and (b) peak ring stage phase for all rhythmic infections, calculated using Maximum Entropy Spectral Analysis (MESA) and METACYCLE. Mice were either WT (C57BL/6J) or clock-disrupted *Per1/2*-null mice with parasites that: were matched to the host’s feeding-fasting rhythm (WT matched), forced to reschedule to align with the host’s feeding-fasting rhythm (WT mismatched & *Per1/2*-null TRF) or experienced arrhythmic hosts (*Per1/2*-null all-day fed). Days 2-6 are considered the pre-peak window of the infections, while days 7-10 are considered the post-peak window.

**
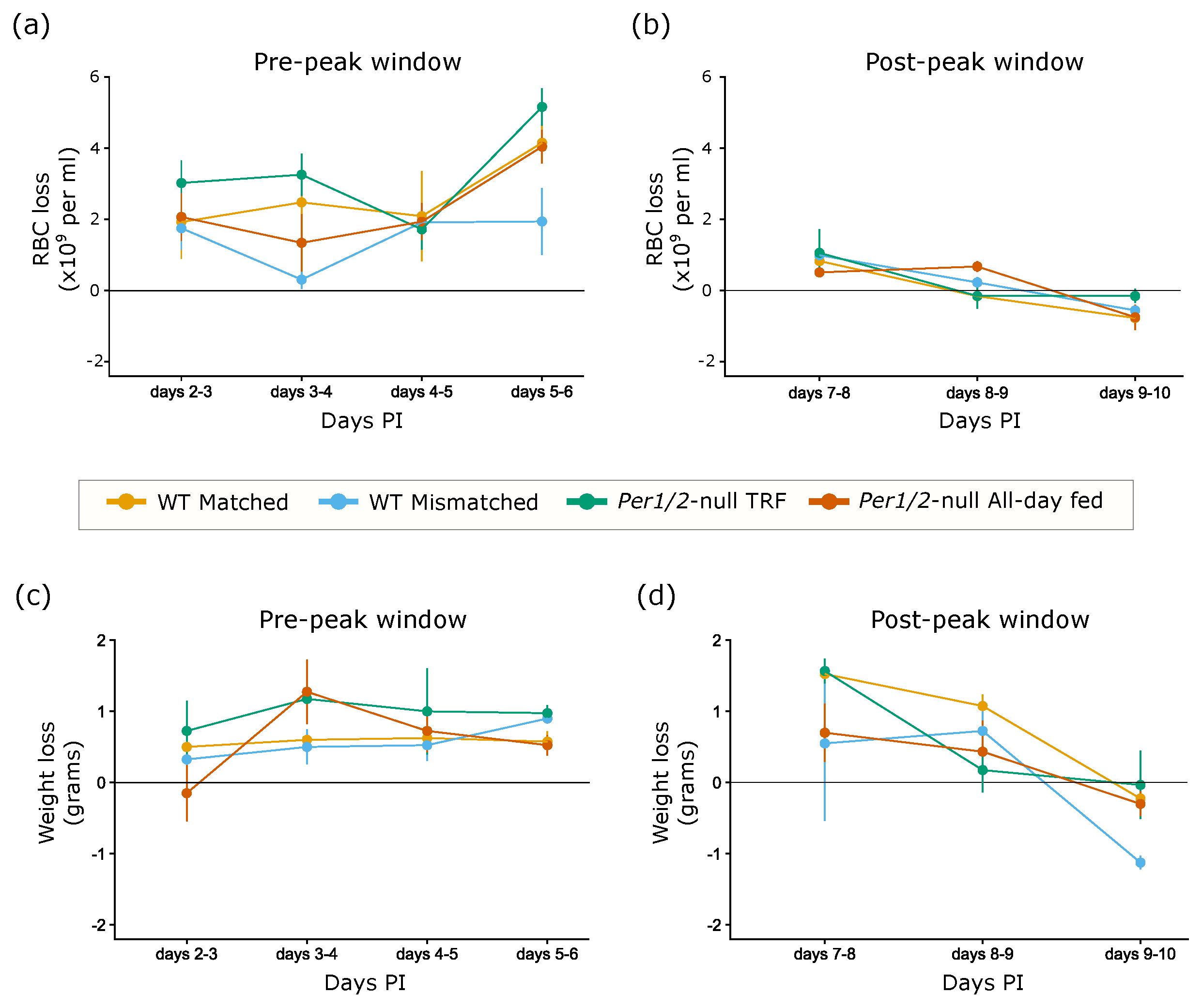
**

**SI Figure 5:** Mean ±SEM (a & b) RBC loss (per ml blood) and (c & d) weight loss, during the pre- and post-peak windows of infection. Hosts were either WT (C57BL/6J) or clock-disrupted *Per1/2*-null mice with parasites that: were matched to the host’s feeding-fasting rhythm (WT matched), forced to reschedule to align with the host’s feeding-fasting rhythm (WT mismatched & *Per1/2*-null TRF) or experienced arrhythmic hosts (*Per1/2*-null all-day fed). a) n = 4 infections per cohort for all groups in the pre-peak window. b) For the post peak window, n = 4 for WT groups, n=3-4 for *Per1/2*-null TRF, and n = 2-4 for the *Per1/2*-null all-day fed group.

|  | **Model description:** |  |  |  |  |  |
| --- | --- | --- | --- | --- | --- | --- |
|  | *log^10^(density) ~* | **df** | **Log(*L*)** | **AICc** | **ΔAICc** | **weight** |
| *Overlap 1* | hpi + treatment | 7 | -10.90 | 37.79 | 0.00 | 0.666 |
|  | hpi * cohort * treatment | 18 | 5.32 | 40.56 | 2.77 | 0.166 |
|  | hpi | 4 | -16.69 | 42.07 | 4.28 | 0.079 |
|  | hpi + cohort + treatment | 8 | -12.12 | 42.87 | 5.07 | 0.053 |
|  | hpi + cohort * treatment | 11 | -9.44 | 45.95 | 8.16 | 0.011 |
|  |  |  |  |  |  |  |
| *Overlap 2* | hpi + treatment | 7 | 12.24 | -8.47 | 0.00 | 0.629 |
|  | hpi | 4 | 7.94 | -7.21 | 1.27 | 0.334 |
|  | hpi + cohort + treatment | 8 | 10.06 | -1.50 | 6.98 | 0.019 |
|  | hpi + cohort | 5 | 6.02 | -1.01 | 7.47 | 0.015 |
|  | hpi * cohort + treatment | 9 | 9.70 | 1.93 | 10.40 | 0.003 |
|  |  |  |  |  |  |  |
| *Overlap 3* | 1 (null model) | 3 | 26.29 | -46.17 | 0.00 | 0.462 |
|  | hpi | 4 | 26.37 | -44.06 | 2.11 | 0.161 |
|  | cohort | 4 | 26.05 | -43.43 | 2.74 | 0.117 |
|  | hpi * cohort + treatment | 9 | 32.00 | -42.68 | 3.50 | 0.080 |
|  | treatment | 6 | 27.92 | -42.37 | 3.80 | 0.069 |
|  |  |  |  |  |  |  |
| *Overlap 4* | treatment | 5 | 14.94 | -17.16 | 0.00 | 0.479 |
|  | 1 (null model) | 2 | 10.17 | -15.86 | 1.29 | 0.251 |
|  | cohort + treatment | 6 | 15.37 | -14.75 | 2.41 | 0.144 |
|  | cohort | 3 | 10.35 | -13.71 | 3.45 | 0.086 |
|  | cohort * treatment | 9 | 20.09 | -12.19 | 4.97 | 0.040 |
|  |  |  |  |  |  |  |
| *Overlap 5* | 1 (null model) | 2 | 12.02 | -19.52 | 0.00 | 0.561 |
|  | cohort | 3 | 12.87 | -18.64 | 0.88 | 0.362 |
|  | treatment | 5 | 13.88 | -14.75 | 4.76 | 0.052 |
|  | cohort + treatment | 6 | 14.86 | -13.30 | 6.22 | 0.025 |
|  | cohort * treatment | 9 | 16.44 | -3.64 | 15.88 | 0.000 |

**SI table 1:** Degrees of freedom (df), log-Likelihood (log(*L*)), AICc, ΔAICc (AICcmodel − AICcmin model), and weight (AICc weight) for each linear model in the analysis comparing parasite densities at each cohort overlap for the rescheduling experiment. Models are in descending order by fit.

|  | **Model description:** |  |  |  |  |  |
| --- | --- | --- | --- | --- | --- | --- |
|  | *Amplitude ~* | **df** | **Log(*L*)** | **AICc** | **ΔAICc** | **weight** |
| *Pre-peak* | treatment | 5 | 42.45 | -73.85 | 0.00 | 0.940 |
| *window* | treatment + days PI | 8 | 43.52 | -68.36 | 5.49 | 0.060 |
|  | 1 (null model) | 2 | 27.43 | -50.67 | 23.18 | <0.001 |
|  | treatment * days PI | 17 | 47.89 | -48.18 | 25.67 | <0.001 |
|  | days PI | 5 | 28.31 | -45.56 | 28.29 | <0.001 |
|  |  |  |  |  |  |  |
| *Post-peak* | treatment + days PI | 7 | 28.40 | -38.13 | 0.00 | 0.987 |
| *window* | treatment | 5 | 20.37 | -28.42 | 9.71 | 0.008 |
|  | days PI | 4 | 18.34 | -27.20 | 10.94 | 0.004 |
|  | 1 (null model) | 2 | 13.66 | -22.91 | 15.22 | <0.001 |
|  | treatment * days PI | 13 | 34.17 | -22.11 | 16.02 | <0.001 |

**SI table 2:** Degrees of freedom (df), log-Likelihood (log(*L*)), AICc, ΔAICc (AICcmodel − AICcmin model), and weight (AICc weight) for the top five linear models in the analysis comparing rhythm amplitude for infections within the rescheduling experiment. Models are in descending order by fit.

|  | **Model description:** |  |  |  |  |
| --- | --- | --- | --- | --- | --- |
|  | *Phase ~* | **#parameters** | **log(*L*)** | **WAIC** | **ΔWAIC** |
| *Pre-peak* | treatment * days PI | 17 | -9.34 | 44.19 | 0.00 |
| *window* | treatment + days PI | 8 | -39.56 | 93.18 | 48.99 |
|  | treatment | 5 | -75.28 | 161.82 | 117.63 |
|  | days PI | 5 | -79.05 | 169.07 | 124.88 |
|  | 1 (null model) | 2 | -95.15 | 194.39 | 150.20 |
|  |  |  |  |  |  |
| *Post-peak* | treatment * days PI | 13 | -33.01 | 87.09 | 0.00 |
| *window* | treatment + days PI | 7 | -39.77 | 92.77 | 5.69 |
|  | treatment | 5 | -42.16 | 93.42 | 6.33 |
|  | 1 (null model) | 2 | -50.12 | 104.91 | 17.83 |
|  | days PI | 4 | -48.87 | 105.79 | 18.70 |

**SI table 3:** Number of model parameters, log-Likelihood (log(*L*)), WAIC, ΔWAIC (WAICmodel − WAICmin model) for the top five Bayesian circular generalized linear models in the analysis comparing rhythm phase for infections within the rescheduling experiment. Models are in descending order by fit.

|  | **Model description:** |  |  |  |  |  |
| --- | --- | --- | --- | --- | --- | --- |
|  | log^10^(density) ~ | **df** | **Log(*L*)** | **AICc** | **ΔAICc** | **weight** |
| *Overlap 1* | *hpi* + cohort + dose | 7 | 23.00 | -29.85 | 0.00 | 0.782 |
|  | *hpi* + dose | 6 | 20.21 | -26.84 | 3.01 | 0.174 |
|  | *hpi* + cohort * dose | 8 | 21.31 | -23.80 | 6.05 | 0.038 |
|  | cohort + dose | 5 | 14.93 | -18.76 | 11.09 | 0.003 |
|  |  |  |  |  |  |  |
| *Overlap 2* | dose | 4 | 40.78 | -72.82 | 0.00 | 0.826 |
|  | cohort + dose | 5 | 40.35 | -69.58 | 3.24 | 0.163 |
|  | *hpi* + dose | 6 | 38.64 | -63.69 | 9.13 | 0.009 |
|  | *hpi* + cohort + dose | 7 | 38.21 | -60.26 | 12.56 | 0.002 |
|  | *hpi* + cohort * dose | 8 | 37.51 | -56.20 | 16.62 | 0.000 |
|  |  |  |  |  |  |  |
| *Overlap 3* | dose | 4 | 40.78 | -72.82 | 0.00 | 0.826 |
|  | cohort + dose | 5 | 40.35 | -69.58 | 3.24 | 0.163 |
|  | *hpi* + dose | 6 | 38.64 | -63.69 | 9.13 | 0.009 |
|  | *hpi* + cohort + dose | 7 | 38.21 | -60.26 | 12.56 | 0.002 |
|  | *hpi* + cohort * dose | 8 | 37.51 | -56.20 | 16.62 | 0.000 |

**SI table 4:** Degrees of freedom (df), log-Likelihood (log(*L*)), AICc, ΔAICc (AICcmodel − AICcmin model), and weight (AICc weight) for each linear model in the analysis comparing parasite densities at each cohort overlap for the dose-dependence experiment. Models are in descending order by fit. For overlap 1, density is best explained by a model including cohort however only 3 of 6 sampling points vary and do so by only 3%.

| **Model description:** |  |  |  |  |  |
| --- | --- | --- | --- | --- | --- |
| *Amplitude~* | **df** | **Log(*L*)** | **AICc** | **ΔAICc** | **weight** |
| 1 (null model) | 2 | -1.58 | 7.60 | 0.00 | 0.580 |
| dose | 3 | -1.18 | 9.29 | 1.69 | 0.249 |
| days PI | 4 | -0.55 | 10.69 | 3.10 | 0.123 |
| days PI + dose | 5 | -0.13 | 12.75 | 5.15 | 0.044 |
| days PI * dose | 7 | 0.47 | 18.15 | 10.56 | 0.003 |

**SI Table 5:** Degrees of freedom (df), log-Likelihood (log(*L*)), AICc, ΔAICc (AICcmodel − AICcmin model), and weight (AICc weight) for each linear model in the amplitude analysis for the dose-dependence experiment. Models are in descending order by fit.

| **Model description:** |  |  |  |  |  |
| --- | --- | --- | --- | --- | --- |
| *Phase~* | **#parameters** | **Log(*L*)** | **WAIC** | **ΔWAIC** |  |
| days PI | 4 | -19.00 | 45.63 | 0.000 |  |
| days PI * dose | 7 | -16.91 | 47.53 | 1.893 |  |
| days PI + dose | 5 | -18.84 | 48.17 | 2.530 |  |
| 1 (null model) | 2 | -27.65 | 59.10 | 13.460 |  |
| dose | 3 | -27.61 | 61.11 | 15.473 |  |

**SI Table 6:** Number of model parameters, log-Likelihood (log(*L*)), WAIC, ΔWAIC (WAICmodel − WAICmin model) for the top five Bayesian circular generalized linear model in the Phase analysis for the dose-dependence experiment.

|  | **Model description:** |  |  |  |  |  |
| --- | --- | --- | --- | --- | --- | --- |
|  | *log10(parasite density) ~* | **df** | **log(*L*)** | **AICc** | **ΔAICc** | **weight** |
| *Pre-peak* | days PI | 5 | -20.45 | 51.01 | 0.00 | 0.997 |
| *window* | treatment + days PI | 7 | -24.25 | 62.72 | 11.70 | 0.003 |
|  | days PI * treatment | 9 | -34.53 | 87.42 | 36.41 | <0.001 |
|  | 1 (null model) | 4 | -126.50 | 261.08 | 210.06 | <0.001 |
|  | treatment | 6 | -130.41 | 272.98 | 221.96 | <0.001 |
|  |  |  |  |  |  |  |
| *Post-peak* | days PI | 5 | 142.24 | -274.28 | 0.00 | 0.997 |
| *window* | treatment + days PI | 7 | 138.57 | -262.75 | 11.53 | 0.003 |
|  | days PI * treatment | 9 | 132.59 | -246.54 | 27.74 | <0.001 |
|  | 1 (null model) | 4 | 91.51 | -174.88 | 99.39 | <0.001 |
|  | treatment | 6 | 87.79 | -163.28 | 111.00 | <0.001 |

**SI Table 7:** Number of model parameters, log-Likelihood (log(*L*)), WAIC, ΔWAIC (WAICmodel − WAICmin model) for the top five linear models in the cumulative parasite density analysis for infections within the rescheduling experiment. Models are in descending order by fit.

|  | **Model description:** | **df** | **Log(L)** | **AICc** | **ΔAICc** | **weight** |
| --- | --- | --- | --- | --- | --- | --- |
|  | *RBC loss ~* |  |  |  |  |  |
| Pre-peak | treatment + days PI | 8 | -1434.67 | 2887.95 | 0.00 | 0.960 |
| window | days PI | 5 | -1441.70 | 2894.43 | 6.48 | 0.038 |
|  | treatment | 5 | -1444.89 | 2900.81 | 12.86 | 0.002 |
|  | 1 (null model) | 2 | -1450.14 | 2904.49 | 16.54 | <0.001 |
|  | days PI * treatment | 17 | -1428.68 | 2904.67 | 16.72 | <0.001 |
|  |  |  |  |  |  |  |
| Post-peak | days PI | 4 | -833.98 | 1677.13 | 0.00 | 0.949 |
| window | treatment + days PI | 7 | -832.68 | 1682.98 | 5.84 | 0.051 |
|  | days PI * treatment | 13 | -827.69 | 1695.94 | 18.80 | <0.001 |
|  | 1 (null model) | 2 | -852.52 | 1709.37 | 32.24 | <0.001 |
|  | treatment | 5 | -852.03 | 1715.87 | 38.74 | <0.001 |
|  |  |  |  |  |  |  |
|  |  |  |  |  |  |  |
|  | *Weight loss ~* |  |  |  |  |  |
| *Pre-peak* | days PI | 5 | -55 | 121.04 | 0 | 0.384 |
| *window* | 1 (null model) | 2 | -58.64 | 121.48 | 0.44 | 0.308 |
|  | treatment + days PI | 8 | -52.04 | 122.69 | 1.66 | 0.168 |
|  | treatment | 5 | -56.01 | 123.06 | 2.02 | 0.14 |
|  | days PI * treatment | 17 | -46.98 | 141.26 | 20.22 | <0.001 |
|  |  |  |  |  |  |  |
| *Post-peak* | days PI | 4 | -55.68 | 120.36 | 0 | 0.782 |
| *window* | treatment + days PI | 7 | -52.94 | 122.91 | 2.56 | 0.218 |
|  | 1 (null model) | 2 | -65.83 | 135.94 | 15.58 | <0.001 |
|  | days PI * treatment | 13 | -49.42 | 136.58 | 16.23 | <0.001 |
|  | treatment | 5 | -64.07 | 139.69 | 19.33 | <0.001 |

**SI Table 8**: Degrees of freedom (df), log-Likelihood (log(*L*)), AICc, ΔAICc (AICcmodel − AICcmin model), and weight (AICc weight) for each linear model in the analysis comparing (top) host RBC loss and (bottom) host weight loss at the end of each cohort (days PI) for the rescheduling experiment. Models are in descending order by fit.
